# Problem-Solving and Behavioural Activation for Young Mothers with Depression in Harare, Zimbabwe: A Mixed-Methods Case Series

**DOI:** 10.3390/epidemiologia6040072

**Published:** 2025-11-03

**Authors:** Concilia Tarisai Bere, Rufaro Hamish Mushonga, Rhulani Beji-Chauke, Patrick Smith, Jermaine Dambi, Dzifa Abra Attah, Takudzwa Mtisi, Dixon Chibanda, Melanie Abas

**Affiliations:** 1 Faculty of Medicine and Health Sciences, University of Zimbabwe, Mt. Pleasant, Harare P.O. Box MP 167, Zimbabwe; rufarohamish@gmail.com (R.H.M.); takudzwamtisi@gmail.com (T.M.); 2 Institute of Psychiatry, Psychology and Neuroscience, Faculty of Social Science & Public Policy, Kings College London, London WC2R 2LS, UK; rhulani.beji-chauke@kcl.ac.uk (R.B.-C.); melanie.abas@kcl.ac.uk (M.A.); 3 Friendship Bench, 4 Weal Road, Milton Park, Harare 0002, Zimbabwe; jermaine.dambi@friendshipbench.io (J.D.); dixon.chibanda@friendshipbench.io (D.C.); 4 Department of Psychiatry, Medical School, University of Ghana, Legon, Accra P.O Box LG 25, Ghana; daattah@ug.edu.gh

**Keywords:** African Youth, depression, anxiety, behavioural activation, problem solving therapy, cultural, contextual adaptation, acceptability, feasibility

## Abstract

**Background.** Depression and anxiety among young people in Africa are highly prevalent and a significant public health concern. Evidence-based interventions (EBIs) tailored to this demographic’s unique cultural and contextual needs are limited. **Methods.** We evaluated an intervention that integrates Behavioural Activation (BA) into Problem-Solving Therapy (PST), focusing on its acceptability, feasibility, preliminary impact on depression and anxiety, and necessary adaptations. Three participants with clinically elevated depression received the six-week intervention. Measures of depression (PHQ-9) and anxiety (GAD-7) were administered pre-intervention and at six subsequent time points. **Results.** PHQ-9 scores decreased from a baseline median score of 15 (Q1–Q3: 11–17) to a follow-up median score of 3 (Q1–Q3: 1–8). GAD-7 score decreased from a baseline median score of 12 (Q1–Q3: 5–14) to a median score of 6 (Q1–Q3: 1–8). Participants endorsed BA components, emphasizing social interaction and achievement-oriented activities, which were perceived as empowering and culturally resonant. Qualitative feedback highlighted the need for adaptations, including simplified language and localized examples, to enhance relevance. **Conclusions.** Findings support the feasibility of task-sharing BA-enhanced PST with lay workers, but point to the necessity of iterative cultural adaptation to address socioeconomic barriers.

## 1. Introduction

Mental health conditions, such as depression, are under-addressed among young people in sub-Saharan Africa (SSA), despite their high prevalence and profound impact on social and economic well-being [1]. In Zimbabwe, approximately 27% of young people report symptoms of anxiety and depression, often associated with intersecting stressors such as unemployment, poverty, and HIV-related challenges [2,3]. While evidence-based psychological interventions, such as problem-solving therapy (PST) [4] and behavioural activation (BA), have demonstrated efficacy in high-income settings with adults and young people [5], their direct application in low-resource settings and culturally distinct contexts, like Zimbabwe, is often limited. This is usually associated with mismatches between therapeutic frameworks and local values, idioms of distress, and help-seeking norms [6,7]. Cultural adaptation of such interventions is critical to improve accessibility, engagement, and outcomes in populations where mental health stigma and reliance on familial or spiritual support systems prevail [8].

The African Youth in Mind Zimbabwe project culturally and contextually adapted the Friendship Bench, an evidence-based intervention that utilises a cognitive behavioural therapy (CBT) based approach at primary care level to address “kufungisisa”, literally meaning “thinking too much” [9]. Originally developed for adults with depression in Zimbabwe, the Friendship Bench utilises PST and has demonstrated efficacy in addressing depression in adults [9]. However, evidence suggests that PST may have limited clinical effectiveness for young people [10]. A trial in Zimbabwe conducted among youth living with HIV highlighted developmental and contextual challenges, as adolescents often struggle with the abstract cognitive demands of PST, which requires complex problem-solving skills and future-oriented thinking [10]. This aligns with the broader literature on qualitative studies of youth with lived experiences, which indicates that youth experiencing depression may benefit more from interventions targeting immediate behavioural engagement rather than PST [11]. More recent evidence from a clinical trial on the delivery of PST to young people indicated greater acceptability for peer-delivered PST [12]. Study recommendations also highlight the need to further adapt peer-delivered PST by adding more active ingredients to enhance its clinical effectiveness [13]. Therefore, we integrated BA following a review of active therapeutic ingredients by the Wellcome Trust and other literature, which emphasizes BA’s strong and promising evidence base for youth with depression [11]. Combining PST with BA creates a dual-focused approach, i.e., PST addresses specific stressors through problem-solving while, BA counteracts the inertia and low motivation characteristics of depression in part by supporting the development of behaviours and activities that are rewarding and reinforcing [14].

PST and BA are particularly promising for young people in Zimbabwe, where pragmatic skill-building aligns with communal values of resilience and collective problem-solving [15]. PST’s focus on actionable strategies to navigate adversity complements BA’s emphasis on modifying maladaptive behaviours, such as social withdrawal or avoidance [4,5]. However, the core tenets of these therapies, such as individualism, explicit emotional disclosure, and the therapist–client hierarchy, may conflict with Zimbabwean cultural norms, which prioritize family interdependence, indirect communication of distress, and respect for elder authority [15]. To address these challenges, this study aimed to adapt BA’s content from the original protocol to include contextually and culturally sensitive values, activities, and homework practices according to the young people of Zimbabwe. We also looked at the intervention’s language, which was translated into Shona (the dominant local language in Zimbabwe), with all worksheets, metaphors, and psychoeducational materials adapted to reflect colloquial language.

We aimed to test the preliminary clinical effects of this novel intervention for young people, ensuring that the intervention is culturally and developmentally appropriate and minimizing the risks of poor uptake or fidelity when implemented at scale. Specifically, in this developmental case series we sought to (1) evaluate the engagement and acceptability of the intervention among young people with depressive and anxiety symptoms; (2) examine changes in depressive and anxiety symptoms over time; and (3) identify key contextual and cultural factors that may inform further adaptation and scale-up of youth-targeted mental health interventions in low-resource settings. Over six weekly sessions, the intervention integrated standard PST-BA techniques, such as problem identification and definition, brainstorming solutions, and solution implementation, and behavioural activation value-related and positive activities [16]. We also added, at the beginning of the sessions, psychoeducation, and, as the last session, relapse prevention. A more detailed outline of the intervention is attached as supplementary material.

## 2. Materials and Methods

### 2.1. The Study Design

We employed an explanatory sequential mixed-methods design, incorporating pre- and post-intervention symptom measures, as well as semi-structured qualitative interviews, to assess initial signs of clinical effect, feasibility, acceptability, and adaptation needs among three young mothers from Zimbabwe (aged 20–23). Participants, recruited from an urban community mental health clinic in Harare, presented with moderate depressive and anxiety symptoms related to relationship pressures, family responsibilities, and socioeconomic stress.

### 2.2. Participants

Participants, presenting with moderate depressive and anxiety symptoms were recruited from an urban community mental health clinic in Harare. They presented with symptoms related to relationship pressures, family responsibilities, and socioeconomic stress. Eleven participants (*n* = 7 males and *n* = 4 females) were screened. Three females between the ages of 20 to 24 years met the criteria for baseline measures and were recruited into the study.

### 2.3. Setting

The study was conducted at Rujeko Clinic, a community health facility in Dzivarasekwa, Harare. Like most high-density suburbs in Zimbabwe, Dzivarasekwa is a densely populated suburb facing socioeconomic challenges, including high unemployment and limited access to mental health services, all contributing to poor mental health among young people [17].

### 2.4. Inclusion Criteria

We screened participants aged 15 to 24 who frequently visit the primary healthcare facilities. Participants under 18 were asked to give informed assent and be willing to involve a caregiver to provide their informed consent. To ensure continued assessment post the intervention period, individuals had to demonstrate a willingness and ability to be followed up over three months. Our main focus was depression, but because depression is highly associated with anxiety, we also assessed for anxiety. However, having elevated symptoms of anxiety was not a requirement for inclusion. Candidates completed the PHQ-9 to assess depression severity, and those scoring 11 or higher were included.

To confirm the diagnosis of depression, an assessment by a qualified mental health specialist using either the DSM-5 or ICD-11 diagnostic criteria was conducted [18]. Ensuring proper clinical diagnosis strengthened the reliability of the study’s findings and ensured that all participants genuinely met the criteria for depressive disorders. Our study participants were recruited through a two-step process. All young people visiting the clinic were asked to give verbal consent to be pre-screened using the Patient Health Questionnaire (PHQ-2), a two-question screening tool for likely depression commonly used in primary healthcare in Zimbabwe [6]. Second, those scoring 2 or higher moved on to the second stage consent process for further assessment and intervention.

### 2.5. Exclusion Criteria

We excluded candidates who were already receiving psychological treatment for any common mental disorder through formal healthcare services. This criterion ensured that the study focused on individuals who were not currently engaged in structured mental health interventions, allowing for a clearer assessment of the novel intervention. Additionally, participants were excluded if they had an advanced physical illness or an active major mental condition, determined by a previous clinical diagnosis of a severe mental health condition that could affect their ability to engage in the study (*n* = 2). This decision was made to ensure participants’ safety and to maintain the study’s focus on individuals who could actively engage with the intervention. Individuals who were actively suicidal, as determined through screening using the P4 screener, were also excluded to prioritize their immediate need for specialized care beyond the scope of the study (*n* = 1). Another exclusion criterion involved sensory impairments that could hinder participation. Specifically, individuals with significant visual or hearing impairments are defined as being unable to see and read the intervention manual or hear the interventionist from approximately one meter away [19]. These impairments were assessed during the informed consent procedures to ensure that all participants could fully engage with the intervention materials and activities. Participants who presented with a PHQ-9 score of below 2 were also excluded from the study as they did not meet the required criteria (*n* = 5).

### 2.6. Measures

The PHQ-9 tool was previously validated against the gold standard, the Structured Clinical Interview of the Diagnostic and Statistical Manual for DSM-IV Axis I Disorders, for use in Zimbabwe [20]. Its optimal cutoff is >11 with a sensitivity for detecting of 85% (95%CI: 78–90%) and specificity of 69% [7]. Additionally, we assessed generalized anxiety disorder (GAD) using the generalized anxiety disorder scale (GAD-7), which was also validated for use in Zimbabwe with a cutoff of >10, a sensitivity of 89% (95%CI: 81–94%), and specificity of 73% (95% CI: 65–80%) [20]. However, this was not a requirement for inclusion.

In this study, scores of 5, 10, 15, and 20 indicated mild, moderate, moderately severe, and severe levels of depression, respectively. Scores of 0–4 were considered minimal [21]. Similarly, the Generalized Anxiety Disorder-7 (GAD-7) is a seven-item self-report scale scored from 0 to 3 for each item (total 0–21) [22]. Established score thresholds categorize anxiety as minimal (0–4), mild (5–9), moderate (10–14), or severe (≥15). This is in line with standard scoring with the two measures [22].

### 2.7. The Intervention

**Figure 1:**
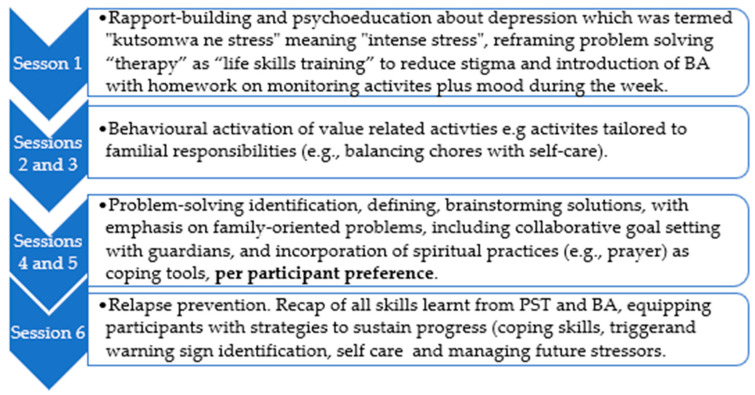
*Overview of the intervention sessions using a culturally adapted psychosocial approach*.

The African Youth in Mind (Y-Mind) intervention included psychoeducation tailored for Zimbabwean youth, the adult PST, and the original BA translated into Shona (Figure 1 below). The psychoeducation was based on contributions made during a two-day stakeholders’ engagement workshop. The workshop participants included a group of young people (15–24 years old), mental health experts, caregivers, community leaders, and lay counsellors. The draft included storytelling of how young people experience depression and anxiety as part of the psychoeducation.

PST is a brief, structured method that helps individuals handle stressful life challenges by teaching systematic problem-solving skills. Instead of dwelling on past experiences, it focuses on clearly defining current problems, generating solutions, making decisions, and evaluating outcomes to enhance coping confidence [4]. In contrast, BA is a short-term therapy aimed at combating depression by encouraging people to re-engage in meaningful and rewarding daily activities [11]. By breaking patterns of withdrawal and avoidance, BA increases exposure to positive reinforcement, which boosts mood and motivation. Studies show it to be as effective as cognitive behavioral therapy (CBT) and antidepressant medications in treating depression, with moderate to large improvements in well-being [16]. The full intervention includes six weekly, 60 min individual sessions conducted by a Zimbabwean Clinical Psychologist trained in PST and BA. These sessions combine evidence-based techniques with culturally responsive adjustments based on pilot feedback from stakeholders and qualitative interviews with young people who have experienced depression, (Figure 1 below). Further modifications were made after this case series, and those results are reported elsewhere.

### 2.8. Qualitative Data

Following the intervention, we conducted semi-structured qualitative interviews to understand participants’ perceptions of cultural acceptability, barriers, and potential modifications to the intervention. The interview guide (attached as supplementary material) concentrated on participants’ experiences with the Y-Mind program, emphasizing what was beneficial and what was not. Inquiries encompassed the two primary components, namely experience with Behavioral Activation (BA) and Problem-Solving Therapy (PST). This included aspects deemed advantageous and aligned with personal values. Additional discussions involved cultural or contextual factors that impact access to care, relationships with counselors, and preferences for counselor attributes, such as age and gender. The guide also explored newly acquired skills or insights, preferred settings for program delivery, and how participants would describe the program to peers. Ultimately, participants were invited to share further reflections or suggestions for future adaptations.

### 2.9. Data Analysis

To analyze our quantitative data, we used descriptive statistics, i.e., frequencies, medians (interquartile range), means (SD), and visualizations (line graphs) to describe participant characteristics and changes in clinical outcomes. To analyze post-intervention interviews, we employed a thematic analysis approach utilizing NVivo software (version 14). Two coders, independently familiarized themselves with the data Via two scripts and coded each transcript. One coder was from Zimbabwe, and the other from the Ghana team, to enhance transparency. The coders met to compare coding, resolve discrepancies, and iteratively refine the codebook. Following the coding process, patterns within the data were organized into preliminary themes. These themes were further refined through an analysis of participants’ perspectives.

### 2.10. Ethical Considerations

We obtained ethics approvals from the King’s College London ethics board (HR/DP-21/22-32917), the Joint Research Ethics Committee (JREC) of the University of Zimbabwe and Parirenyatwa Group of Hospitals (Ref JREC/300/22), and the Medical Research Council of Zimbabwe (Ref: MRCZ/A/2965), approval date 6 December 2023. Safety protocols included immediate referral to psychiatric services for participants presenting with suicidal ideation during the study; this was performed through the clinic manager. Anonymity was maintained using participant codes, and caregiver assent for participants aged <18 was secured prior to participation.

## 3. Results

### 3.1. Clinical Observations

Below, in Table 1, Table 2 and Table 3 we report the results from the clinical observations, assessments performed using the PHQ-9, GAD-7, and the qualitative interviews.

Clinical Observation: The intervention was conducted by a qualified clinical Psychologist to learn the mechanisms of therapy and areas that needed to be adapted in the intervention protocol.

Table 1 below presents the clinical observations for each participant.

**Table 1:** *Participant 1 clinical observations*.

Participant 1: Persistent Depression Linked to Familial Trauma
Demographics: A 24-year-old single woman from Dzivarasekwa Harare, urban setting, not employed with a 2-year-old girl child.
Presenting Concerns: The participant reported moderate depressive symptoms (PHQ-9 score: 15/27). Symptoms included persistent low mood, anhedonia, insomnia, and suicidal ideation. Her distress was rooted in different family, financial problems and the abduction of her younger brother two weeks prior. At the time of the meeting, the participant had no resolution despite police efforts. She described overwhelming guilt, (“I should have protected him”) and rumination about his potential death. The client thought that taking her life was an option so she could meet her brother in heaven. Suicide risk assessment was performed, and although she had thoughts of ending it all, the risk for suicide was low.
Intervention and Progress: During six weekly sessions of the Y-Mind intervention, she engaged minimally initially, expressing hopelessness. In session 2, she chose to go to the police to help them with the search for her brother. In session three, she went away to spend time with her aunties, who live close to their house, which she found helpful. Session 4 focused on problem-solving family relationships and financial issues. Her assertiveness to help the police helped, as in session four, she reported having found her brother, who had been located alive, though traumatised. By session 5 and 6, her PHQ-9 score dropped to 5 then 3, reflecting improved mood and renewed engagement in social and pleasurable activities. She attributed her recovery to both the intervention’s coping strategies (e.g., problem-solving and performing activities that gave her a sense of achievement) and the resolution of her brother’s case, stating, “Knowing he’s safe let me breathe again.” At 6-week follow-up, her PHQ-9 score stabilised with no active suicidal ideation. She resumed vocational training, highlighting the interplay between external stressors and therapeutic support in her trajectory.

**Table 2:** *Participant 2 clinical observations*.

Participant 2: Resilience Amidst Financial and Relational Stressors
Demographics: A 23-year-old single mother of a 3-year-old, living in a low-income neighbourhood.
Presenting Concerns: Moderate depression (PHQ-9: 17/27) linked to family relationship conflict and financial instability. She reported arguments with her mother and grandmother over household responsibilities and debt. Despite stressors, she emphasised a “strong faith in God” and a goal to become a Master of Ceremony at her church.
Intervention and Progress: In Y-Mind sessions, she utilised positive activities from the onset to manage anxiety, and she learned new problem-solving skills to address conflicts with her family. Notably, her mood improved markedly around Easter (PHQ-9: 1/27 at week 4), coinciding with her active role in church events. She described Easter services as “a reminder that I have purpose beyond my struggles.” While financial strain persisted, her mood gains were sustained through increased social participation (e.g., choir practice which was BA activity chosen through homework practice) and reframing challenges as “temporary tests.”
Outcome: Post-intervention, her PHQ-score was 1 and stayed low. She began mentorship under her church’s lead conductor, aligning with her aspirational identity. Her case underscores the protective role of goal-directed problem solving, social positive behaviour and community engagement in buffering depressive symptoms.

**Table 3:** *Participant 3 clinical observations*.

Participant 3: Trauma Recovery Through Empowerment and Advocacy
Demographics: A 20-year-old single mother of a three-year-old, unemployed, with a history of intimate partner violence (IPV).
Presenting Concerns: Moderate depression (PHQ-9: 11/27) and PTSD symptoms stemming from a 5-year abusive relationship. She faced ongoing legal battles for child custody and financial support, which exacerbated helplessness (“I’m trapped in his shadow”).
Intervention and Progress: Initial Y-Mind sessions focused on safety planning and emotional regulation particularly for this participant because of the trauma they had experienced. Her grandmother helped the participant to cope, and her family was concerned about her safety. After session 3, we referred her to the Msasa Project, a gender-based violence support organization, which provided legal advocacy. Concurrently, she launched a small hair-braiding business under a tree at her grandmother’s house, securing income and rebuilding self-efficacy. This decision was made as a result of problem-solving strategy planning. By session 6, her PHQ-9 decreased to 6, with noted reductions in hypervigilance and improved problem-solving (“I can fight for my child and feed her”).
Outcome: At the 6-week follow-up, PHQ-9 was 4/27. Though court proceedings continued, her business thrived, and she joined a survivor advocacy network. This case highlights the importance of integrated support where there might be the risk of IPV. Survivors can combine psychological intervention, economic empowerment, and systemic advocacy.

### 3.2. Quantitative Results

**Figure 2:**
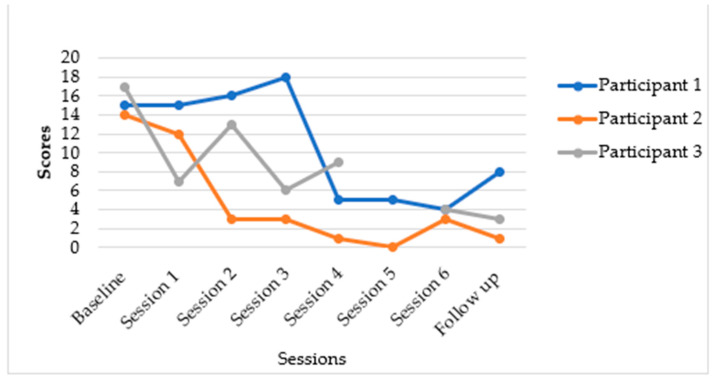
*Changes in PHQ-9 scores over time*.

**Figure 3:**
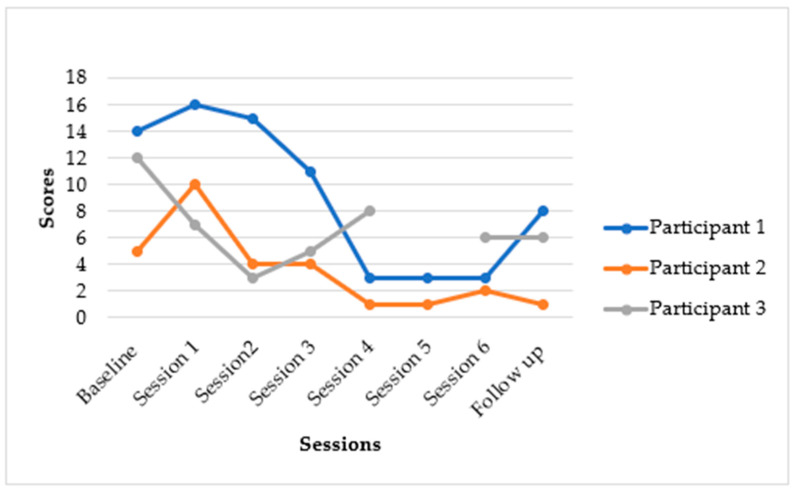
*Changes in GAD-7 scores over time*.

Figure 2 and Figure 3 below indicate the results from the PHQ-9 and GAD-7 assessments. Depression symptoms gradually decreased over time, with PHQ-9 scores decreasing from a baseline median score of 15 (Q1–Q3: 11–17) to a follow-up median score of 3 (Q1–Q3: 1–8). Anxiety symptoms on the GAD-7 decreased from a baseline median score of 12 (Q1–Q3: 5–14) to a median score of 6 (Q1–Q3: 1–8) at follow-up.

The Y-Mind intervention demonstrated notable reductions in depressive and anxiety symptoms, though with some variability in the extent of individual participant improvement. Meaningful changes in depression for most participants were observed.

### 3.3. Individual Participant Outcomes for Depression (PHQ-9) and Anxiety (GAD-7)

The following summarises the individual participants’ results: As indicated in Figure 2, participant one presented with moderate depressive symptoms, reporting a PHQ-9 score of 15/27. Her distress was linked to familial trauma and financial problems, including the recent abduction of her younger brother. The participant’s PHQ-9 score dropped to 5 and then 4. This represented a substantial reduction of 11 points from baseline (15 to 4). This participant moved from a moderately severe depression category to a minimal depression category. Participant two’s baseline score indicated that she had moderate depression, with a PHQ-9 score of 17/27, associated with family relationship conflict and financial instability. At follow-up, her mood improved markedly, with her PHQ-9 score dropping to 1/27 by session 4, and these mood gains were sustained post-intervention. These scores indicated a significant reduction of 16 points from baseline (17 to 1). This participant seemed to have transitioned from a moderately severe depression category to a minimal depression category. Participant 3 (20-year-old single mother):

Participant three’s baseline score showed that she presented with moderate depression and PTSD symptoms, with a PHQ-9 score of 11/27, stemming from a history of intimate partner violence. The participant’s results for session 5 are missing because the participant had an appointment for a physical ailment, and we continued with her in session 6. Sessions 5 and 6 covered similar content; therefore, the participant was able to continue with session 6 despite having missed session 5. At follow-up, her PHQ-9 score decreased to 4 by session 6, and remained at 3/27 at the 6-week follow-up. This demonstrated a reduction of 8 points from baseline (11 to 3). Overall, depression symptoms gradually decreased over the course of the intervention. Anxiety (GAD-7): Anxiety symptoms also showed a considerable decrease. Generalised Anxiety Disorder Scale-7 (GAD-7) score for all participants reduced from a baseline of 12 (Q1–Q3: 5–14) to a median score of 6 (Q1–Q3: 1–8) at follow-up.

### 3.4. Qualitative Results

Our qualitative study results mirror the quantitative findings in Figure 2 and Figure 3. Participants found this intervention to be acceptable and feasible. Participants in this developmental case series described the Y-Mind intervention as a turning point in their ability to manage depression well and re-engage meaningfully with their daily activities. They highlighted how Y-Mind fostered social connections, emotional relief and renewed hope. The young mothers reported that structured and purposeful activities such as attending church, social activities, and sports like netball and football helped them improve mood, re-establish social connections, and reduce isolation. This resonates with findings from other studies on youth mental health interventions which indicate that regular physical activity improves mental health by reducing symptoms of depression through enhancing self-esteem and strengthening social skills [23]. In addition, participants highlighted that physical activity alleviated psychological stress, which helped with suicidal ideation [23]. Participants also valued PST as it helped them identify, understand, and address problems associated with stress. The young mothers expressed feelings of relief after trying out the intervention’s homework-related activities. However, concerns about trust and confidentiality arose when participants had to share their problems with non-family members. This highlights the need for enhanced safeguards and trust-building measures. Taken together, our findings suggest that even in contexts marked by socio-economic challenges, culturally adapted psychosocial interventions like the Y-Mind can alleviate problems associated with depression among young people.

## 4. Discussion

The Y-Mind developmental case series evaluated a novel psychological intervention tailored for youth in Zimbabwe, demonstrating substantial reductions in depression and anxiety symptoms. These reductions not only reflect significant improvements, but also clinically meaningful change, moving participants from moderate to minimal symptom ranges. Such outcomes point to the intervention’s potential to address the growing mental health burden among youth. The marked symptom reductions suggest that the intervention was both engaging and acceptable to this population. The youth-centric design element, such as culturally adjusted interactive modules, likely enhanced adherence and receptivity. By aligning with young people’s preferences for cultural activities that involve family, the intervention may have mitigated traditional barriers to care (e.g., stigma and disengagement).

Furthermore, the transition to subclinical symptom levels may imply tangible benefits for daily functioning, including improved occupational, social, and emotional well-being. A key driver of the intervention’s acceptability may lie in its integration of behavioural activation (BA) strategies, which directly engage youth in goal-directed activities that foster social interaction and personal achievement [16]. By structuring sessions around collaborative tasks (e.g., family-oriented challenges, creative projects) and individualized goal setting (e.g., mastering a skill, re-engaging in hobbies), the intervention likely empowered participants to counter avoidance and isolation, standard features of depression and anxiety [24]. The emphasis on small, achievable steps provided the young people with immediate reinforcement through successes, aligning with their developmental need for a sense of achievement and mastery [25]. This approach not only made the intervention feel less clinical, but also cultivated intrinsic motivation by linking effort to tangible, rewarding outcomes (e.g., improved mood, peer and family bonding).

Evidence from sub-Saharan Africa further supports this, with a study in Zimbabwe showing that peer-led counselling for adolescents with HIV—modified to include problem-solving within structured problem discussion therapy to better align with young people’s relational difficulties and limited agency—led to measurable improvements in symptoms of common mental disorders [10]. Although this intervention did not improve virological suppression compared to standard peer support, its success was in its adaptability to focus on problem discussion rather than executing specific tasks, underscoring the importance of customizing therapeutic approaches to developmental and contextual factors [10]. Conversely, other research, including a scoping review and an exploratory meta-analysis, indicates that problem-solving training alone may not significantly lower depressive symptoms among youth aged 14 to 24 years [11]. The review, which looked at four randomized controlled trials, found a small and statistically non-significant effect (Hedges’ g  =  −0.34, 95% CI: −0.92 to 0.23), with high heterogeneity and low-quality evidence [11]. These findings suggest that while PST might help youth address personal challenges, those with high depressive symptoms may require more comprehensive psychotherapeutic support beyond PST. This supports our reason for combining PST with BA, as BA can target motivation issues and social withdrawal that PST alone may not sufficiently address.

In Kenya, the Shamiri intervention—a school-based, layperson-led group program focused on character strengths like growth mindset, gratitude, and value affirmation—showed larger reductions in depressive symptoms immediately after treatment, at 2 weeks, and at 7 months follow-up [26]. It also led to greater decreases in anxiety at posttreatment and the 7-month mark, compared to an active study skills control [26]. These positive effects persisted for at least 7 months, demonstrating the long-lasting impact of culturally relevant, school-based methods for young people in resource-limited settings. Notably, participants found both the Shamiri sessions and the study skills control to be beneficial [26]. This suggests that acceptability may depend more on delivery style and cultural relevance than on the specific therapeutic content [26]. Our qualitative data support this, as young people described Y-Mind activities as productive and helpful for distracting them from negative thoughts, indicating that interventions perceived as valuable and relevant promote ongoing engagement and symptom improvement. 

Findings from other settings highlight the importance of activation and structured, skills-based work in improving youth outcomes [11]. Brief behavioral therapy that emphasizes activation and exposure achieves greater clinical improvement than assisted referral, especially among underserved populations [11]. This demonstrates how activation-focused content can lead to meaningful change when delivered in an accessible manner [11]. Overall, these studies support the plausibility of our observed symptom improvements and our qualitative feedback on acceptability. They also suggest that integrating behavioral activation into a culturally adapted problem-solving approach may be particularly effective for adolescents, who benefit from small, achievable steps that offer immediate reinforcement, enhance social connections, and foster mastery. These results align with regional and global evidence that brief, developmentally appropriate, and culturally sensitive interventions can reduce internalizing symptoms in youth when delivered by trained non-specialists in routine settings [11].

The qualitative reports from the exit interviews suggest that participants valued these activities as “productive” and “distracting from negative thoughts,” highlighting their role in enhancing engagement and perceived relevance. Furthermore, participants expressed positive feedback about the intervention, highlighting its role in promoting mental well-being and providing a supportive space to navigate personal challenges. The young mothers also found the culturally specific intervention to be useful in meeting their needs. Both spiritual and medical explanations of their experiences were addressed. This is in line with results from other qualitative studies conducted in sub-Saharan Africa [27]. Many described how the intervention helped them build resilience, regain hope, and develop healthier coping mechanisms. The personal counts illustrate the profound impact of the sessions in transforming participants’ lives, from overcoming distress to finding renewed purpose and motivation.

### 4.1. Practical Implications

The findings of this study underscore significant considerations for the design and implementation of mental health interventions aimed at young women. The integration of PST and BA demonstrated effectiveness in reducing symptoms of anxiety and depression, indicating that comprehensive approaches may be especially effective in addressing the multifaceted challenges encountered by this demographic. Concurrently, the study highlights the necessity for cultural and contextual adaptation. Interventions should not be presumed to operate uniformly across varied settings. Customizing content and delivery to align with local realities and values is essential to ensure relevance and acceptability.

Our qualitative findings underscore the importance of incorporating lived experience voices in the development of interventions. Involving young people directly in the design ensures that strategies are culturally sensitive, which can increase engagement. While these results offer promising evidence of feasibility and impact, future research should use more rigorous methods and larger, more diverse samples. Rigorous studies are essential to strengthen the evidence and assess how well these adapted interventions can be scaled across various cultural and community environments.

### 4.2. Limitations

Several limitations warrant consideration when interpreting the findings of this case series, particularly concerning the adaptation process and its generalizability at this stage. The cultural and contextual adaptation process was conducted exclusively with three female single mothers aged 20–24 years. This was performed after a few attempts to include males and younger adolescents but at the time most did not meet recruitment criteria. Specifically, the male individuals who were under 18 were excluded due to a high risk of suicide. While the demographic of young mothers represents a high-priority group facing significant challenges in Dzivarasekwa, their specific lived experiences, priorities, and socio-cultural context (e.g., motherhood responsibilities, financial pressures, relationship dynamics unique to young single mothers) inherently shaped the resulting adaptations. Consequently, the adaptations at this stage reflect the needs, preferences, and contextual realities of young single mothers in this setting. As these adaptations cannot be equally suitable or resonant for other young people within the broader target population of 15–24-year-olds (such as adolescent males or females, non-parents, young parents in partnered relationships, or individuals from slightly different socio-cultural backgrounds), more work needs to be performed. Developmental stages, life contexts, communication styles, and key concerns (e.g., school, parental dependence, and identity formation) might differ between mid-to-late adolescents (15–17 years) and young adults (20–24 years). Further adaptations to the intervention and rigorously testing it with young people across all ages between 15 and 24 are essential.

## 5. Conclusions

Combining PST and BA offers a foundation for developing accessible and acceptable mental health interventions for youths. Nonetheless, its initial promise must be validated through rigorous testing. Future iterations should include detailed cultural and contextual adaptations while preserving the core active components that enhance engagement. Key next steps involve conducting a randomized controlled trial to evaluate effectiveness, gathering feedback from more young people to assess acceptability and mechanisms of change, and addressing implementation aspects like cost-effectiveness and optimal platforms for delivery to ensure real-world applicability. This work aids the creation of innovative, scientifically robust interventions that are highly responsive to the needs of young people in low-resource settings, where the demand is high and research remains limited.

## Data Availability

The original contributions presented in this study are included in the article/supplementary material. Further inquiries can be directed to the corresponding author.

## Supplementary Materials

The following supporting information can be downloaded at: https://www.mdpi.com/article/10.3390/epidemiologia6040072/s1, Table S1: Case series qualitative interview; Table S2: Cultural adaptations of PST and BA.

## Abbreviations

The following abbreviations are used in this manuscript:

BA	Behavioural Activation
PST	Problem Solving Therapy
PHQ-9	Patient Health Questionnaire-9
GAD 7	Generalised Anxiety Disorder 7
IPV	Intimate Partner Violence
Y-mind	Youth in mind
EBI’s	Evidence Based Interventions
SSA	Sub-Saharan Africa
HIV	Human immunodefiency virus
