# Supplementary material for: Problem-Solving and Behavioural Activation for Young Mothers with Depression in Harare, Zimbabwe: A Mixed-Methods Case Series
Source: Epidemiologia (Basel). 2025 Nov 3;6(4):72. doi: 10.3390/epidemiologia6040072 (PMC12641939; doi:10.3390/epidemiologia6040072)
Supplement: Supplementary file 1 [file epidemiologia-06-00072-s001.zip › Table S1-Case series qualitative interview.pdf]

1. Interviewer: The reason why we came here is to find out how the Y-Mind intervention has helped you. We would want to hear your experience on what helped you and what didn't help you. So my first question is that can you explain your experiences receiving the Y-Mind intervention.
2. Can you explain what you liked about the intervention?
3. Do you have anything you didn't like about the intervention.
4. So you started with Behavioural activation where you were asked about activities that could make you happy and fulfilled. What did you like?
5. How did the activities help you?
6. Any challenges you faced doing the activities?
7. Ok, so how did the activities align with your values?
8. What can be improved about BA so that it can help young people?
9. The second part of the intervention was Problem Solving Therapy where you were asked about challenges that you were facing. Can you explain what you liked about it.
10. What did you not like?
11. What challenges did you face doing those Problem Solving?
12. what do you think can be done to improve Problem Solving?
13. Looking at Behavioural Activation and Problem-Solving Therapy, what components was more helpful? Explain why?
14. Looking at cultural and contextual issues, is there anything that affected you to access adequate care? Whether you have been hindered by a relative or something
15. How was your relationship with your counsellor?
16. Interviewer: Did you feel free and helped?
17. What was not helpful looking on you relationship.
18. What needs to be changed looking at our counsellors and the relationship?
19. What age do you think the counsellor should be? Should she be a man or woman and of what age?
20. Do you still find the intervention you received still useful up to now.
21. What new things did you learn about the Y-Mind intervention?
22. Participant: I learnt that, I must do activities. I must not loose power. Even if l have a challenge which
23. Where do you think is the best place to carry out this intervention?
24. So if you meet young people like you about the Y-Mind intervention. What would you say?
25. Do you still have anything more to say about Y-Mind?
