# Supplementary material for: Problem-Solving and Behavioural Activation for Young Mothers with Depression in Harare, Zimbabwe: A Mixed-Methods Case Series
Source: Epidemiologia (Basel). 2025 Nov 3;6(4):72. doi: 10.3390/epidemiologia6040072 (PMC12641939; doi:10.3390/epidemiologia6040072)
Supplement: Supplementary file 1 [file epidemiologia-06-00072-s001.zip › Table S2-Cultural adaptations of PST and BA.pdf]

Cultural adaptations of PST and BA (Y-MIND Zimbabwe)

Manual content	Domain	Cultural adaption
Depression symptomology	Low mood	Description: “Feeling like I can’t do anything and my parents calling me lazy”
	Loss of interest	Description: “No longer doing the things I could do like fending for self”
	Social isolation	Description: “staying in my room and my family getting worried” (aligning with local norms of hospitality)
	Fatigue	Description: “Body heavy like I can’t do anything at home” (referencing daily chores)
Psychoeducation	Depression	Kutsomwa ne stress *(overburden by stress)*: to describe emotional heaviness<br>Kuita kupwanyika pwanyika so *(confusing/not easy to understand loss of energy)*: to describe fatigue<br>Kurikitwa ne stress *(thinking too much)*: to describe rumination
	Struggles	Kugara mugomba rine rima *(to sit in a dark pit)*: to frame helplessness that sometimes leads to suicide<br>Kune mwenje kuseri kumugero *(there’s light at the end of this dark tunnel)*: used in cases of seeking help i.e., at the Youth in Mind
Behavioral activation	Communal chores and home responsibilities	Activity: framed chores (fetching water, farming, cooking) as “caring for the family” rather than individual tasks<br>Metaphor: “a single broomstick cannot sweep the homestead” (proverb emphasizing collective effort)
	Health and relationships	Group activities: structured BA around HIV support groups (common stressor) or church youth gatherings<br>Trauma integration: used storytelling circles to link activities to resilience (“how did your grandmother cope during hard times?”
	Traditional games	Revived games: replaced social media with group games like *nhodo* (stone counting) or *chamu hwande hwande* (hide-and-seek), emphasising social reconnection
	Church gatherings	Spiritual alignment: integrated prayer/music into goal-setting (if acceptable to the participant)
Problem-solving therapy	Resolving family conflicts	Practiced “speaking respectfully to elders” using culturally scripted dialogues (“how to ask for study time instead of chores”)<br>Metaphor: “a family is like a baobab tree – strong when roots work together”

	Securing financial aid	Solutions: Brainstormed communal strategies (group savings clubs [mukando], selling crafts at church)
	Trauma context	Acknowledged shame around poverty, sexual and physical abuse ("many struggles that we face that feel like you are facing an elephant that you have to eat, let's find a way forward together")
	Peer pressure	Scenarios: addressed local issues (pressure to engage in transactional sex for money, selling substance skin lightening scream and marijuana)<br>Language: used youth slang ("kutomborara", "kutsomwa" vs risky behaviors)
	Conceptual shifts	Reframed mental health: avoided clinical terms; PST called "building strengths/skills for life's puzzles"<br>Collective grieving: normalised trauma via shared narratives ("we've all lost someone – let's learn to carry that weight")
Relapse prevention	Local symbols of resilience	Metaphor: "like the mbira music that survives drought, you've learned rhythms to keep your spirit strong"<br>Activity: youth created "resilience maps" using local landmarks ("my strength is like Zambezi river – always following")
	Community anchors	Plan: identified community supports (auntie, teacher, church lead) to turn to during setbacks<br>Ritual: designed a closing "handing over the light/certificate" ceremony with a shared laugh in some cases song
	Trauma-sensitive coping for a few clients	Strategies: taught grounding techniques using familiar sensory cues ("focus on the smell of soil after rain")
	Relapse language	Reframed setbacks: normalised struggles as "the path having stones, not the walker being weak"
